# Cancer-Associated Arterial Thrombosis: Mechanisms and Risk Factors

**DOI:** 10.32604/or.2026.074452

**Published:** 2026-04-22

**Authors:** Kassiani Lalechou, Despoina Pantazi

**Affiliations:** Laboratory of Biochemistry, Faculty of Chemistry, School of Sciences, University Campus, University of Ioannina, Ioannina, Greece

**Keywords:** Anticancer drugs, cancer-associated arterial thromboembolism, pathophysiology, risk factors, thrombosis

## Abstract

Cancer-associated thrombosis (CAT) is a leading cause of morbidity and mortality among cancer patients. While venous thromboembolic events have been extensively studied due to their higher incidence, arterial thrombosis in cancer patients—referred to as cancer-associated arterial thromboembolism (CA-ATE)—is less well understood but may pose a greater danger. The pathophysiology of CA-ATE involves complex interactions between the tumor microenvironment, cancer cells, patient-related factors, and cancer therapies. Some chemotherapeutic agents, particularly platinum-based compounds (cisplatin, oxaliplatin), gemcitabine, taxanes, and targeted therapies such as tyrosine kinase inhibitors (TKIs), have been associated with an increased risk of arterial thrombosis. In certain patient populations, hormonal therapy and selective estrogen receptor modulators may also contribute to this risk. Additionally, factors such as patient age, cancer type, and stage contribute to an increased risk of arterial thrombosis in this population. Key mechanisms driving CA-ATE include endothelial injury, hypercoagulability, and platelet activation. Certain malignancies, notably lung and pancreatic cancers, are associated with a higher incidence of arterial thrombotic events. The aim of this review is to enhance understanding of the underlying mechanisms of cancer-related arterial thromboembolism and to highlight the various therapeutic, cancer-related, and patient-related factors that contribute to the occurrence of cancer-associated arterial thrombotic events.

## 1 Introduction

Thrombosis is the second leading cause of death in cancer patients, after cancer itself [1,2]. Cancer patients are at high risk of developing either venous thromboembolism (VTE) or arterial thromboembolism (ATE) [1,2]. The first report linking cancer to thrombosis dates back to 1865, when Armand Trousseau described the association between thrombosis and undiagnosed malignancy [3]. Cancer-associated thrombosis (CAT) remains one of the leading causes of non-cancer-related morbidity and mortality among cancer patients [4]. Both tumor microenvironment and anticancer therapies influence hemostatic mechanisms, contributing to thrombotic risk [2,4].

The tumor microenvironment is an important contributor to thrombosis [5]. However, in many patients, thrombosis may be the first clinical manifestation of cancer, often diagnosed within six months of the thrombotic event [6]. Patients with cancer face the risk of both venous and arterial thrombosis, which is more commonly associated with solid tumors but can also include disseminated intravascular coagulation [6]. Venous thromboembolism has been extensively studied, as it is the most frequent thrombotic complication in cancer patients [7]. Recently, however, there has been a notable increase in ATEs, attributed in part to newer chemotherapeutic agents. This underlines the need for further investigation into the mechanisms and risk factors underlying CA-ATE [7]. Several strategies have been implemented in clinical practice to prevent CAT. These include anticoagulant therapy with low-molecular-weight heparin (LMWH) or direct oral anticoagulants, with individualized risk assessment to balance the benefits of thrombosis prevention against the risk of bleeding and close monitoring according to patient condition, cancer type and treatment regimen [8]. Risk stratification is usually performed using validated tools, such as the Khorana score, which incorporates tumor type, blood counts, and Body Mass Index (BMI) to identify patients at higher risk of VTE [9]. The American Society of Hematology (ASH) 2021 guidelines recommend primary thromboprophylaxis with low-molecular-weight heparin (LMWH) or direct oral anticoagulants (DOACs, e.g., apixaban, rivaroxaban) for high-risk ambulatory patients, while suggesting DOACs or no prophylaxis for intermediate-risk patients [10]. The International Initiative on Thrombosis and Cancer (ITAC) 2022 guidelines similarly endorse prophylaxis for high-risk patients, particularly those with pancreatic cancer or a Khorana score ≥2 [11]. The European Society for Medical Oncology (ESMO) 2023 guidelines extend recommendations to all ambulatory cancer patients with a predicted 6-month VTE risk ≥8%–10% [12]. The British Society for Hematology (BSH) 2024 specifically recommends thromboprophylaxis for pancreatic cancer patients and considers it for other high-risk malignancies [13]. Despite these recommendations, prophylaxis remains inconsistently applied in clinical practice due to concerns about bleeding risk and treatment complexity [14]. Importantly, no formal guidelines currently exist for the prevention of CAT, highlighting a major gap in evidence-based management. The aim of this review is to provide a comprehensive overview of cancer-associated arterial thromboembolism, focusing on its underlying pathophysiological mechanisms and the key cancer-related, treatment-related, and patient-related risk factors contributing to its development.

## 2 Epidemiology

In 2022, nearly 20 million people were newly diagnosed with cancer worldwide, and around 10 million deaths were attributed to the disease. Projections based on population growth suggest that by 2050, the number of annual new cancer cases could reach 35 million, marking a substantial increase from current levels [15]. Given this high burden of cancer worldwide, complications such as thrombosis represent a major contributor to mortality. It is widely recognized that thrombosis is the second most common cause of death in cancer patients after cancer itself [1,2,16]. In fact, patients with active cancer are 4 to 7 times more likely to develop CAT [4,17]. Research data indicate that patients with diagnosed malignancy account for 20%–30% of VTE cases in the general population [17]. Over the last 20 years, studies have shown an increase in the 12-month incidence of VTE, rising from 1% in 1997 to 1.9% in 2004 and 3.4% in 2017 [16,18].

As mentioned, VTE can present as the first symptom of undiagnosed cancer in asymptomatic patients. Specifically, 4% of individuals who develop VTE for the first time will be diagnosed with malignancy within the following 6 months [16,18]. Furthermore, within 6 months of starting cancer treatment, the risk of VTE increases 12- to 23-fold compared to the general population [17]. This risk is even higher for patients receiving chemotherapy or targeted therapies, with 15%–25% experiencing recurrent thrombosis [16–18].

Cancer patients also have an increased risk of ATE, including myocardial infarction (MI) and stroke [6]. Although arterial thrombosis has been less extensively studied than venous thrombosis, ATE occurrence is linked to poorer treatment response, higher risk of recurrent thromboembolism (37% within the next 6 months), and increased mortality [19]. The cumulative incidence of ATE within 6 months of cancer diagnosis ranges from 1% to 5% [4].

To investigate ATE incidence, Navi et al. conducted a large retrospective study using data from the Surveillance, Epidemiology, and End Results (SEER) database [20]. The study analyzed 279,719 pairs of newly diagnosed cancer patients and matched controls from 2002 to 2011 [20,21]. Cancer types included breast, prostate, bladder, non-Hodgkin lymphoma, lung, stomach, and pancreatic cancers [21]. Within the first 6 months after diagnosis, ATE incidence was 4.7% in cancer patients vs. 2.2% in controls [21].

Among cancer types, patients with lung, stomach, and pancreatic cancer had higher risks of ATE, with incidence rates of 8.3%, 6.5%, and 5.9%, respectively. However, ATE incidence significantly decreased after one year, regardless of cancer type [21]. Advanced cancer stages were associated with higher ATE risk; for example, 7.7% of patients with stage 4 cancer developed ATE within 6 months of diagnosis, compared to 2.3% of patients with stage 0 cancer [21].

MI occurred more frequently (3.0% of cancer patients) than ischemic stroke (2.0% within 6 months post-diagnosis) [21]. Notably, patients who experienced acute ischemic stroke had a high and progressively increasing rate of recurrent ATE—21% at 1 month, 31% at 3 months, and 37% at 6 months [19].

A recent meta-analysis by Deng et al., which included data from over 5 million patients, reported an incidence of ATE of 11.60 per 1000 person-years. Cardiac events such as MI (6.11 per 1000 person-years) and stroke (9.07 per 1000 person-years) constituted a significant proportion of these events [22].

After surgery, patients have an increased likelihood of arterial thrombosis, such as infarction or ischemic stroke, due to a combination of tissue injury, inflammation, metabolic changes and hemostasis disturbances [23,24]. Anesthesia may also contribute through temporary fluctuations in pressure and coagulation [25]. The fact that the increased risk is mainly limited in the early period after surgery suggests that surgery mainly activates an already existing thrombogenic state in the body. In a large national registry studied by Navi et al., approximately 1% of patients with disseminated cancer experienced perioperative MI or ischemic stroke, with a significant mortality of 20% [26]. Similarly, Rautiola et al. followed the cumulative incidence of MI and ischemic stroke in the first year after surgery [25]. The results showed that the cumulative incidence of MI was 1.33% and ischemic stroke was 1.25%, with respective ORs of 1.28% and 1.25% compared with the reference population [25]. Events were more severe when recorded during hospitalization for surgery (e.g., PCI or bypass), whereas after hospital discharge, rates of serious or fatal events remained similar [25].

## 3 Pathophysiology

### 3.1 Venous Thrombosis

Venous thrombosis includes both deep vein thrombosis and pulmonary embolism, which are initiated by a valve in the blood vessels [27]. Among the predisposing factors for thrombosis are reduced blood flow, low shear stress, and limited oxygenation [27]. Platelets and leukocytes trapped in the valve sites increase thrombotic activity [27]. Thrombosis in the veins is mainly fibrin-rich and is produced when the endothelium is damaged and the coagulation system is activated by tissue factor (TF) activation [28]. The mechanisms in CAT are described recently by several investigators [2,29]. The coagulation cascade causes prothrombin to be converted into thrombin, leading to platelet aggregation at the site of injury [28].

### 3.2 Arterial Thrombosis

Pathogenesis of arterial thrombosis is primarily due to Virchow’s triad endothelial damage [27,30]. The main cause is the instability of atherosclerotic plaques, which occurs when the plaques are rich in lipids and have a thin fiber cap, making them susceptible to thrombotic events [27]. When the plaques rupture, thrombus formation is stimulated by activation of the platelets [27]. This is due to the high rate of shear stress in the narrowed arteries, since platelets are the only cellular components that can stick and activate in these conditions [29]. High shearing intensities actually promote platelet activation [29]. Although arterial thrombosis in cardiac patients is usually due to the rupture of atherosclerotic plaques, in cancer patients it is less likely to be related to underlying atherosclerosis [29]. More specifically, systemic hypercoagulability driven by tumor-derived factors, such as thrombin and vascular endothelial growth factor (VEGF), may also be involved in ATE [29]. These factors contribute to the activation of platelets and therefore to thrombosis without necessarily the rupture of atherosclerotic plaques [29].

Platelets are one of the major regulators of hemostasis [31,32]. They have a short lifespan of no more than 10 days and are removed to maintain platelet homeostasis, with a new 10^11^ platelets daily production [31]. Upon intravascular injury, platelets activated and interacted with the coagulation cascade, leading to clot formation [33].

Platelets play a key role in progression, metastasis and, more generally, in arterial thrombosis associated with cancer [34]. Their cancer-related hyperactivity has been observed in a variety of tumor types, including endometrial and cervical carcinomas [35]. Common features of this hyperactivity are reduced survival, reduced prostacyclin sensitivity, and increased levels of soluble products such as platelet factor 4 (PF4) and thrombospondin [35]. *In vitro*, tumor cells have been shown to activate platelets either through direct contact or by releasing stimulants such as ADP, thromboxane A2 (TXA2) and thrombin, which promote tumor-associated procoagulation [35]. The mechanism of tumor cell induced platelet aggregation (TCIPA) is particularly important, since it is induced in various types of cancers such as colorectal, renal, and pancreatic cancer [34]. ADP produced by cancer cells, induces platelet aggregation via the P2Y1 and P2Y12 receptors, whereas thrombin activates platelets via the PAR-1 and PAR-4 receptors. Finally, activation of platelets contributes to metastasis [2,36]. Thromboxane A2 also enhances this process, and both GPIIb-IIIa and GPVI receptors play a central role in platelet adhesion to neoplastic cells and the vascular wall [36]. In parallel, overexpression of the Major Histocompatibility Complex class I (MHC-I) helps cancer cells evade recognition by natural killer (NK) cells and enhances their migratory capacity [37].

Although increased levels of vWF and enhanced platelet aggregation via ADP have been recognized as factors promoting thrombosis, the exact clinical relevance of these events is still under investigation [35]. Platelet microparticles (PMPs) excreted during platelet activation also play an important role in angiogenesis, metastasis and thrombosis and provide a platform for the transport of TF and phospholipids [38,39]. Increased levels of these PMPs have been reported in colorectal and breast cancers [4,38,40].

Simultaneously with platelet activation, the coagulation cascade is also triggered, involving both the intrinsic (contact activation) and extrinsic (TF) pathways, which partially overlap [41].

The initiator of the extrinsic coagulation pathway is TF, which is expressed by endothelial cells at the site of vascular injury [41]. The complex formed between activated factor VII (FVIIa) and TF (FVIIa-TF) is responsible for activation of FX, which is converted to FXa in the presence of Ca^2+^ [42]. Synergies between FVa and FXa activate prothrombin, which is converted to thrombin [32,42].

The intrinsic coagulation pathway is initiated by factor XII (FXII), a zymogenic serine protease [43]. FXII becomes activated (FXIIa) upon contact with negatively charged surfaces such as polyphosphate (poly-P), which is released by activated platelets [44]. FXII can also interact with exposed endothelial collagen, though this typically occurs only upon rupture of an atherosclerotic plaque [41]. Activation of FXII is further enhanced by a synergistic mechanism involving prekallikrein and high-molecular-weight kininogen (HMWK) [33]. FXIIa subsequently catalyzes the conversion of factor XI (FXI) to FXIa. FXIa, with calcium ions (Ca^2+^) and activated factor VIII (FVIIIa), promotes the activation of factor IX (FIX to FIXa) [43]. FVIIIa is synthesized primarily in the liver and endothelial cells, circulates in plasma bound to von Willebrand factor (vWF), and becomes active following vascular injury [33]. Upon endothelial disruption, the FVIII-vWF complex dissociates, allowing FVIIIa to participate in the intrinsic tenase complex with FIXa, ultimately leading to activation of factor X (FX) to FXa [33].

At this stage, the intrinsic and extrinsic coagulation pathways converge, as the FIX–FVIIIa complex from the intrinsic pathway cooperates with the TF–FVIIa complex from the extrinsic pathway to activate factor X (FX) to FXa [33,41]. FXa, in conjunction with activated factor V (FVa), calcium ions (Ca^2+^), and negatively charged phospholipid surfaces, forms the prothrombinase complex, which catalyzes the conversion of prothrombin to thrombin [33]. Thrombin plays a central role in the coagulation cascade by converting fibrinogen into fibrin [33]. Simultaneously, thrombin exerts positive feedback on the cascade by activating FV, FVIII, and FXI [33]. All mechanisms for arterial thrombosis are summarized in Table 1 and described with details subsequently.

**Table 1:** Overview of molecular mechanisms implicated in arterial thrombosis in cancer patients.

Molecular/Cellular Pathway	Key Components/Mechanisms	Clinical Association/Impact	References
Platelets	Activation by high shear stress via ADP (P2Y1/P2Y12), Thrombin (PAR-1/PAR-4), Thromboxane A2, GPIIb-IIIa & GPVI	Platelet aggregation, contributes to the metastatic process, and enhances arterial thrombosis	[27,29,31–36]
Tumor Cell-Induced Platelet Aggregation (TCIPA)	Direct contact or secretion of ADP, TXA2, Thrombin by cancer cells	Increased Risk of thrombosis in various cancers (e.g., pancreatic, renal, colorectal)	[34–36]
Platelet Microparticles (PMPs)	Tissue Factor (TF) and phospholipid carriers, involvement in angiogenesis and metastatic process	Increased risk of thrombosis, involvement in cancer types such as breast and colorectal	[4,38–40]
Coagulation Cascade—Extrinsic Pathway	TF + FVIIa → FX → FX → FXa → Prothrombin → Thrombin	Potential contribution to thrombosis without necessarily rupturing plaques	[2,19,32,41,42]
Coagulation Cascade—Intrinsic Pathway	FXII → FXIIa → FXI → FXIa → FIX → FIXa → Tenase complex (with FVIIIa & vWF) → FX → FXa	Contribution to thrombin, cooperation with exogenous pathways for thrombosis	[33,41,43,44]
vWF & FVIII	FVIII transport, endothelial activation	Enhancement of platelet adhesion and accumulation by high shear stress	[21,31,33,47]
Tumor-derived factors	Thrombin, VEGF, TF, Poly-P from activated platelets	Prothrombotic environment, enhancing platelet activation-driven ATE	[21,29,35]

Note: Abb.: Thromboxane A2 (TXA2), Activated Factor VIII (FVIIIa), Factor X (FX), von Willebrand Factor (vWF), Vascular Endothelial Growth Factor (VEGF), Arterial thromboembolism (ATE).

### 3.3 Direct Mechanisms

#### 3.3.1 TF

TF is a 47 kDa transmembrane glycoprotein that plays a central role in initiating the extrinsic coagulation pathway and contributes significantly to the pathogenesis of CAT [2,17]. Under physiological conditions, TF is expressed by subendothelial cells and becomes active following vascular injury or inflammatory stimuli. Upon activation, TF binds to circulating FVII, converting it to its active form (FVIIa), which in turn activates FX, ultimately leading to thrombin generation and fibrin clot formation [19].

In cancer, TF is often overexpressed on the surface of tumor cells, and it is also released in association with microparticles (MPs) derived from tumor (TMPs) or host cells [2,16]. These TF-bearing MPs disseminate procoagulant activity systemically, increasing the risk of venous thromboembolism. The severity of thrombosis is correlated with the level of TF expression and may range from asymptomatic events to clinically significant and even life-threatening thromboembolic complications [19].

While TF is a well-established mediator of hemostasis and arterial thrombosis under normal and inflammatory conditions, its role in CA-ATE remains unclear. Although some evidence suggests a potential involvement, there are currently no definitive mechanisms linking TF expression directly to the development of arterial thrombotic events in cancer patients [2]. This uncertainty may partly reflect fundamental differences between venous and arterial thrombosis, as arterial thrombus formation is primarily platelet-driven and occurs under high shear stress conditions, where the contribution of TF-dependent fibrin generation may be secondary [45,46]. In addition, most available evidence linking TF to CAT originates from venous thromboembolism studies or experimental models, whereas direct clinical data supporting a causal role of TF in CA-ATE remain limited [4].

#### 3.3.2 von Willebrand Factor

von Willebrand factor (vWF) is a multimeric glycoprotein composed of repeating structural domains, each of which serves a specific functional role [31,33]. These domains act as binding sites for platelet receptors and subendothelial components, including GPVI and collagen [31,33]. vWF, in its inactive form—typically under low shear stress—adopts a compact, globular conformation and primarily functions to protect factor VIII (FVIII) from proteolytic degradation [33]. Upon exposure to high shear rates, vWF undergoes conformational changes that activate its adhesive functions. Notably, the higher the multimer size, the more potent its role in platelet adhesion and aggregation [33].

vWF is synthesized primarily by endothelial cells and megakaryocytes. While megakaryocyte-derived vWF is incorporated into platelets, only endothelial-derived vWF contributes to platelet adhesion under physiological flow conditions [33]. In cancer patients, plasma levels of vWF are often elevated. This is partly attributed to direct production by tumor cells, which may aberrantly express and secrete vWF [47].

Moreover, elevated vWF levels not only reflect endothelial dysfunction or damage but also actively contribute to thrombogenesis by enhancing platelet–subendothelial matrix interactions [21]. Inflammatory cytokines such as tumor necrosis factor-alpha (TNF-α) and interleukin-1 (IL-1), which are frequently elevated in malignancy, further upregulate vWF synthesis and secretion. As a result, cancer patients are predisposed to arterial thrombotic events due to both elevated vWF concentrations and enhanced functional activity [21].

The activation of factor Xa and thrombin can be directly inhibited by oral anticoagulants (DOACs), reducing fibrin formation and thrombus development in VTE [48]. This direct action provides a predictable and rapid anticoagulant effect, which is associated with lower rates of recurrence and minor bleeding events [49]. In contrast, low-molecular-weight heparins (LMWH) act indirectly through antithrombin, which inhibits factor Xa [50]. Due to variability of renal clearance, LMWH dosing is less predictable [51]. Clinical studies have shown that both drug classes have comparable rates of recurrence and major bleeding, while the more consistent action of DOACs may explain the reduced incidence of minor bleeding [52,53]. Arterial thrombosis (ATE) is primarily mediated by platelets, supporting the use of antiplatelet agents such as aspirin appropriate [46,54]. Aspirin exerts its effect by irreversibly inhibiting platelet function through acetylation of COX-1, thereby blocking the synthesis of thromboxane A2, a molecule that promotes platelet aggregation [55]. However, the risk of bleeding must also be considered, which is elevated in cancer patients due to bone marrow dysfunction or chemotherapy [54,55]. While insights from VTE demonstrate the effectiveness of targeting coagulation pathways, the lack of robust clinical data on ATE in cancer patients highlights the need for further studies to clarify optimal preventive and therapeutic strategies [54].

### 3.4 Indirect Mechanisms

#### 3.4.1 NETs

Neutrophil extracellular traps (NETs) are web-like structures composed of decondensed chromatin DNA, neutrophil granule proteins, and histones, released by activated neutrophils in a process known as NETosis [4,56]. Initially described in 2004, NETs were identified as antimicrobial structures involved in innate immunity and host defense [47,56]. Over the past two decades, however, accumulating evidence has demonstrated a critical role for NETs in the pathophysiology of CAT [4,47].

NET formation can be triggered by interactions with activated platelets via P-selectin, or by inflammatory cytokines such as TNF-α, IL-8, and G-CSF [4,47]. Once released, NETs serve as procoagulant scaffolds by trapping platelets and coagulation factors, and by promoting TF expression and thrombin generation, thereby contributing to a hypercoagulable state [27,57]. Current knowledge of the interaction between platelets and neutrophils and their role in arterial thrombosis has emerged only recently [58]. NET-derived histones can directly activate endothelial cells and increase the expression of vWF, which further enhances platelet adhesion and coagulation [17,27].

While the contribution of NETs to venous thrombosis in cancer patients is studied, their exact role in arterial thrombosis remains less clearly defined [59]. Nonetheless, several studies have reported elevated levels of NET biomarkers—including citrullinated histone H3 (H3Cit), myeloperoxidase (MPO), and G-CSF—in patients with cancer-associated ischemic stroke, suggesting a shared prothrombotic mechanism [59]. These findings underscore the importance of further elucidating NET-driven pathways in arterial thrombosis, especially given the frequent underdiagnosis of cancer in patients with stroke and the implications for treatment strategies.

#### 3.4.2 MPNs

Myeloproliferative neoplasms (MPNs) are a group of clonal hematopoietic stem cell malignancies characterized by the overproduction of one or more myeloid lineages, including erythrocytes, leukocytes, and platelets [60]. MPNs are broadly categorized into BCR-ABL-positive and BCR-ABL-negative neoplasms. BCR-ABL-negative MPNs—including polycythemia vera (PV), essential thrombocythemia (ET), and primary myelofibrosis (PMF)—are more commonly associated with thrombotic complications, which are the leading cause of morbidity and mortality in these patients [60].

A key driver mutation in BCR-ABL-negative MPNs is the JAK2V617F gain-of-function mutation, which leads to constitutive activation of the JAK-STAT signaling pathway and clonal proliferation of myeloid cells [59]. Notably, JAK2 is expressed not only in hematopoietic cells but also in endothelial cells, suggesting the involvement of a common progenitor and underscoring the complex interplay between the vascular and hematopoietic systems in MPN-associated thrombosis [60].

The prothrombotic phenotype in JAK2-mutated MPNs is multifactorial. Firstly, the hyperviscosity caused by erythrocytosis in polycythemia vera contributes to reduced blood flow and increased thrombotic risk [60]. Secondly, JAK2 activation enhances platelet-endothelial interactions by upregulating P-selectin, which promotes platelet adhesion and leukocyte recruitment to the endothelium. Additionally, JAK2 signaling promotes thrombin generation. This, in turn, increases phosphatidylserine (PS) exposure, providing a procoagulant surface that amplifies thrombin production and clot formation [60].

## 4 Risk Factors

Thrombosis in cancer patients is the result of complex interactions between the characteristics of the individual patient, the malignancy itself and the anticancer therapy [61].

### 4.1 Patient Characteristics

**Figure 1:**
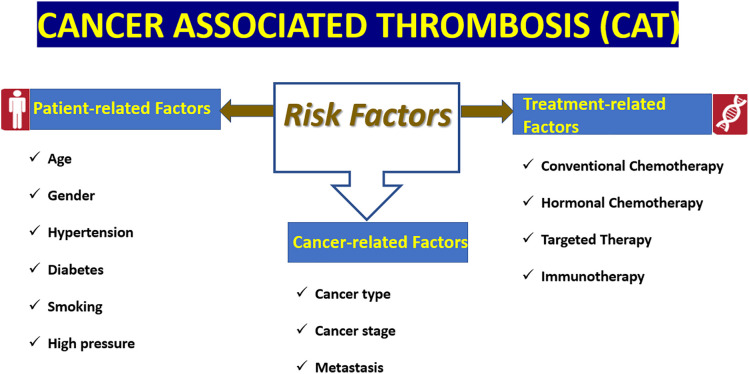
Risk factors for cancer-associated arterial thromboembolism (CA-ATE). CAT-ATE development is influenced by complex interactions between patient factors (e.g., age, gender, hypertension, diabetes, smoking), tumor-related factors (type of tumor, stage of tumor) and anticancer therapy (inhibitors of VEGFR, e.g., cisplatin, tamoxifen, bevacizumab, and BCR-ABL). VEGFR: Vascular endothelial growth factor receptor.

Age is one of the most important predictors of arterial and venous thrombotic events, as it is associated with prolonged immobility and enhanced coagulation activation [29]. Specifically, arteries become stiffer and less elastic, with increased collagen and calcium deposition, and endothelial function decreases, leading to reduced production of protective molecules such as prostacyclin and nitric oxide [62]. In the blood there is an increase in coagulation factors (V, VII, VIII, IX, fibrinogen) and von Willebrand factor, together with a decrease in fibrinolytic activity due to high levels of PAI-1 [63–65]. Platelets become more sensitive and easier to activate, enhancing thrombin production [66]. Reduced mobility and lack of regular exercise led to venous stasis, further increasing the risk of thrombosis [66,67]. In Denmark, a cohort study was conducted in 2021 where patients diagnosed at age 65–75 years had a 1.5-fold increased risk of developing acute thrombocytopenia, which increased to 1.9 when the patient was diagnosed at age 75 years or older [68]. These analyses also took into account the competing risk of death from other causes [68]. Similarly, a study has shown that being 65 years of age or older is an equally important risk factor for thromboembolic events [69] (Fig. 1).

In 2006 a study by Chew et al., gender was not found to be predictive of thrombotic events in any cancer type [70]. In other studies, although women appeared to have a higher rate of VTE, this did not appear to be of major consequence [29]. However, in the aforementioned Danish study [68], a 15% increased risk of arterial thrombosis was observed in men (HR [hazard ratio] 1.15, 95-CI 1.08–1.22). In addition, in the same study [68], major risk factors included hypertension (HR 1.29, 95-CI 1.37) and diabetes (HR 1.20, 95-Ci 2.77–3.17). This is also confirmed by a recent study by Madauto and colleagues [71] in which female patients with CML treated with tyrosine kinase inhibitors (TKIs) had a higher rate of venous thromboembolic events than male patients (6.0% vs. 0.0% *p* = 0.02). Conversely, men with these characteristics are more likely to develop arterial thrombosis than women (23% vs. 9%, *p* = 0.02) [71]. However, there was no significant difference in the incidence of thromboembolic events between men and women (35% male, 32% female *p* = 0.02) [71]. Moreover, arterial hypertension and diabetes were particularly important predictors, particularly in females [71] (Fig. 1).

### 4.2 Cancer-Related Factors

As regards risk factors associated with malignancy itself, the stage of cancer at the time of diagnosis is also of major importance [29]. Two large population-based studies conducted in the United States and Denmark reported HRs of 3.6 and 1.2, respectively, for ATEs associated with metastatic disease [20,68] (Fig. 1).

Furthermore, certain cancer types are more strongly associated with thrombotic complications [20,68]. Pancreatic cancer represents one of the strongest predictors of ATE, with a 4.8-fold increase reported in a Danish study [68] and a 2.5-fold increase in a US cohort [20]. Similarly, lung cancer is associated with a significantly increased risk of ATE, with a 3.8-fold increase reported in Denmark [68] and a 3.5-fold increase in the US [20]. These findings were further supported by the study of Morath et al. [72], which reported an ATE incidence of 5.8% among patients with advanced lung cancer.

Importantly, the association between cancer and thrombosis appears to differ between arterial and venous thrombotic events [20,68]. For instance, although colorectal cancer is not considered a major risk factor for VTE, it has been associated with hematological abnormalities, including platelet alterations, which may contribute to arterial thrombotic risk [20,68]. Although not fully elucidated, these differences are likely attributable to variations in tumor biology as well as to differences in therapeutic approaches across cancer types [20,68].

In addition, patients with lung and gastrointestinal cancers frequently present with cardiovascular comorbidities, such as atrial fibrillation (AF) [29], which may partially explain the increased incidence of arterial thrombotic events observed in these populations; however, further clarification is required [29]. A meta-analysis by Deng et al. confirmed that the incidence of arterial and venous thrombosis is directly associated with tumor location [22]. Specifically, a higher incidence of ATE was observed in patients with lung cancer, gastrointestinal stromal tumors, brain cancer, and bladder cancer, whereas patients with pancreatic, ovarian cancer, and lymphoma exhibited a higher incidence of venous thrombosis [22].

More recent data from the study by Mitrovic et al. [73] highlighted a strong association between ATE and acute myeloid leukemia (AML). The overall incidence of arterial thrombotic events within the first six months following diagnosis was 2.9%, with most events occurring during the first month, particularly in patients with active disease [73]. Mortality in this population was notably high, with approximately one in two patients affected by these events dying [73].

Additional risk factors include traditional cardiovascular conditions such as hypertension, diabetes mellitus, and dyslipidemia [73]. Patients presenting with multiple cardiovascular risk factors exhibited a cumulative ATE incidence of 5.6%–12.5% within two years following cancer diagnosis [73]. Smoking also represents a significant contributor to thrombotic risk, as it is associated with endothelial dysfunction and increased platelet counts [72]. Importantly, smoking cessation has been shown to confer particular benefit in women with lung cancer [72] (Fig. 1).

### 4.3 Treatment-Related Factors

In addition to tumor site and stage, other important risk factors include therapeutic interventions that significantly increase the risk of thrombosis [60]. Chemotherapy is the primary treatment for several malignancies, and chemotherapeutic agents have anti-neoplastic effects by both cytotoxicity and inhibition of cellular processes that are required for the proliferation of malignant cells [74]. However, in addition to targeting malignant cells, cytotoxicity is induced in non-malignant cells, which may contribute to the development of undesirable cardiovascular disorders [74]. The pathogenic mechanisms of anti-cancer agents and their toxic effects on the cardiovascular system have been previously described [75].

#### 4.3.1 Conventional Chemotherapy

Platinum-based chemotherapy is a key treatment option for various cancers, such as ovarian, bladder, cervical, and testicular carcinoma [76]. Cisplatin is the first-generation platinum-based medicine, while newer analogues have been developed [77]. They are widely used in first-line and adjuvant therapy regimens after radiotherapy or surgery [78]. Their anti-cancer activity relies on their ability to form complexes with DNA, impairing replication, and transcription, thereby causing cell death [76]. However, treatment with platinoids is associated with an increased risk of thrombotic complications. Cisplatin, as a first-generation therapy, is associated with an increased risk of venous thromboembolism. Although less toxic in terms of other adverse reactions, carboplatin offers no clear benefit in thrombogenesis compared to cisplatin [79,80]. Oxaliplatin, on the other hand, is associated with a lower incidence of thrombotic complications, suggesting that it may be different from other platinum-based therapies [81,82]. The exact mechanisms by which platinum-based compounds contribute to the prothrombotic environment are not fully understood. Studies have shown that cisplatin-induced cell exposure increases the activity of TF, particularly in immune cells, and induces apoptosis in endothelial cells, releasing extracellular vesicles [82,83]. Cancer cells also produce increased vesicles when exposed to platinum-based compounds, but these have no discernible effect on forming a prothrombotic environment [84]. Clinical data indicate that these cytotoxic therapies induce vWF, platelet activation, and general coagulation system activation [85].

**Figure 2:**
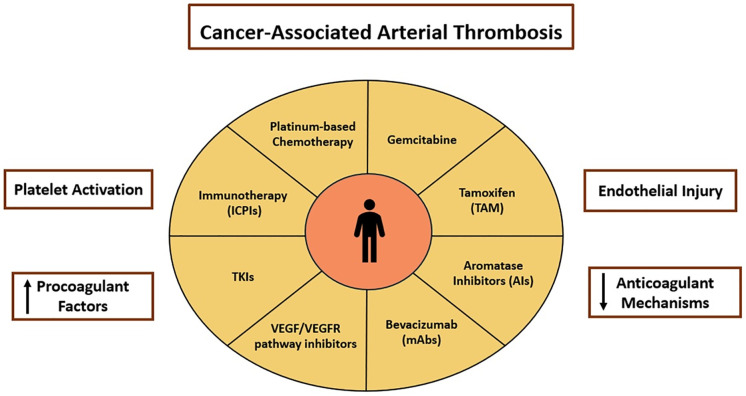
Treatment-related factors for cancer-associated arterial thrombosis (CA-ATE). Platinum-based therapy, gemcitabine, tamoxifen (ΤΑΜ), aromatase inhibitors (AIs), bevacizumab, VEGF/VEGFR (Vascular Endothelial Growth Factor/(Vascular Endothelial Growth Factor Receptor) pathway inhibitors, TKIs (tyrosine kinase inhibitors), Immunotherapy (ICPIs). All these factors can cause platelet activation, increase procoagulant factors, induce endothelial injury and decrease anticoagulants mechanisms.

Other clinical factors associated with these therapeutic approaches, such as dehydration and electrolyte disturbances (hypomagnesaemia), can increase thrombogenesis [86]. Although most studies provide clear evidence of venous thrombosis, arterioles are less well studied, and evidence is mainly provided by case reports and some retrospective studies. With limited data on arterial thrombosis, retrospective studies and case reports are crucial to highlight the rarity and severity of arterial thrombosis events. In one retrospective study involving 932 patients with various types of cancer who were treated with cisplatin, the incidence of thromboembolic events was particularly high, at 18.1%, up to 4 weeks after treatment [87]. In previous studies, the incidences of cerebrovascular accident and MI were not considered thromboembolic events but cardiovascular events, and they were limited to 8.4%–12.9% [87]. Similarly, Weijl et al. investigated 179 patients with genital cancer which showed that of the total thrombotic events, 8.4% were arterial events, including thrombosis of the brain and the lower limbs [88]. Numico and colleagues studied 10 patients with non-small cell lung cancer stage III-IV, where venous thrombosis was observed in 17.6% of all 10 events involving arterial events (MI, cerebral ischemic, and arterial thrombosis of the lower limbs) [89] (Fig. 2).

Gemcitabine is a synthetic, pyrimidine-related antimetabolite with broad use in the treatment of solid tumors such as pancreatic, non-small cell lung, bladder, breast, and ovarian carcinoma [90]. It belongs to a wider class of chemotherapeutic substances that act as purine and pyrimidine analogues by interfering with the synthesis of DNA and the division of cells, predominantly in the S phase [91].

Clinical data indicate that gemcitabine has an effect on hemostatic balance, although the mechanism is not fully understood. Altered platelet function and number due to gemcitabine can induce thrombocytopenia and thrombocytosis, which interfere with normal coagulation pathways and promote a prothrombotic environment [92]. Furthermore, it is likely to cause damage to endothelium, and the immune complexes formed during treatment exacerbate this damage [93,94]. Meta-analyses of randomized studies suggest a trend of 2.1- to 2.2-fold increased risk of thromboembolic events in patients treated with gemcitabine compared with other therapeutic options [95]. However, it should be noted that the total incidence is underestimated since many cases are asymptomatic [95]. Gemcitabine has been associated with thrombotic microangiopathy, a severe complication with high mortality, although it is rare (0.1%–0.3%) [93]. In addition, acute coronary syndrome has been reported, which highlights the potential for serious arterial events [91] (Fig. 2).

#### 4.3.2 Hormonal Chemotherapy

Tamoxifen is a selective estrogen receptor modulator (SERM) used as a chemotherapy agent in patients with estrogen receptor-positive breast cancer [96]. It appears to increase susceptibility to thrombotic events through complex mechanisms which are not fully understood. The effect on platelets appears to be the most important mechanism [96]. In particular, tamoxifen (TAM) has been shown to increase the influx of calcium into platelets, a process that is necessary for their activation, and to increase the aggregation and multiplication of microparticles that are dependent on TF [96]. However, the overall effect on platelets is questionable, as various studies have shown inhibition of thrombin and collagen adhesion and aggregation, leading to reduced arterial clot formation in experimental models [97]. Although the available data are contradictory, several studies have shown reductions in the levels of antithrombin and proteins C and S and have shown resistance to activated protein C [98–100]. In addition, it is associated with increased levels of coagulation factors such as FVII, FXI, and von Willebrand factor, which contribute to thrombogenesis [99] (Fig. 2).

Administration of this cancer drug appears to be associated with an increased incidence of ATE compared with control treatment and other treatments. In particular, there was a significant increase in breast cancer incidence in premenopausal women, 1.6% in comparison with the control group [101].

In a recent meta-analysis [102], 4465 adverse events were reported, including 385 thromboembolic events associated with tamoxifen (TAM). Several important findings were highlighted: an increased thromboembolic risk was observed in men over 85 years old, with an 11.92-fold higher risk compared to men under 45 years old, and in patients weighing more than 80 kg, who had a 2.71-fold higher risk [102]. New thromboembolic signals were also identified, such as monoplegia (ROR [Reporting Odds Ratio] = 7.94), retinal artery occlusion (ROR = 10.80), and atrial thrombosis (ROR = 6.12) [100]. Although women accounted for 78.7% of the cases, the adjusted risk was 2.97 times higher in men, particularly for arterial events such as coronary artery occlusion (ROR in males = 851.62) [102].

Aromatase inhibitors (AIs) such as letrozole, anastrozole, and exemestane are a class of medicines that target the production of estrogens, thereby reducing estrogen levels in postmenopausal women [103,104]. They act by inhibiting aromatase, an enzyme that converts androgens into estrogens in peripheral tissues such as the skin, breast tissue, and bone [103]. Inhibition of aromatase results in significant reductions in estradiol and estrone, thereby reducing the stimulation of estrogenic tumors expressing estrogens [103]. Compared to SERMs such as tamoxifen, aromatase inhibitors offer a more targeted effect with fewer adverse effects on other tissues such as the womb and have been shown in experimental and clinical models to reduce breast tumor growth and progression [103]. Use of aromatase inhibitors in breast cancer patients has been associated with an increased risk of cardiovascular events, especially thrombotic events related to the arteries, such as MI and ischemic stroke [105] (Fig. 2).

The first key mechanism involves the loss of endothelial protection by estrogens, resulting in endothelial dysfunction, impaired lipid metabolism, and enhanced inflammatory response, all of which promote atherosclerosis and arterial thrombosis [103,105]. The second mechanism is related to prothrombotic alterations in the coagulation system, including increased platelet activation and enhanced clotting activity, which, in combination with endothelial dysfunction and systemic inflammation, amplifies the risk of arterial thrombosis [104,105].

In a study of breast cancer patients by Khosrow-Khavar et al. (2020), initiation of aromatase inhibitors (AIs) therapy was associated with an approximately 86% increased risk of heart failure and a 50% increased risk of cardiovascular mortality [104]. In addition, AIs have been linked to a higher incidence of dysrhythmias, valvular dysfunction, and pericarditis compared with tamoxifen, which may confer cardiovascular benefits through improvement of lipid profiles [105,106]. The incidence of cardiovascular events in AIs-treated patients ranges from 1.1% to 60.6%, with an overall incidence of approximately 13%, and high-grade events occurring almost exclusively in AIs users [105].

#### 4.3.3 Targeted Therapy

Bevacizumab is a monoclonal antibody (mAb) that reduces the expression of VEGF, thereby inhibiting angiogenesis [74]. This had been approved in various advanced cancer types, such as non-small cell lung cancer (NSCLC), colorectal, ovarian, kidney and cervical cancer [74]. A large meta-analysis showed an association between bevacizumab and an increased risk of thromboembolic events in the arteries [107]. The severity of these events is dose-dependent and refers to hypertension, cardiovascular events, and ischemic brain injury [107]. Moreover, the type of episodes differs according to the type of tumor [107]. In particular, renal cell carcinoma is associated with increased arterial events, prostate cancer with increased cardiovascular events and breast cancer with increased blood pressure [107]. The mechanisms for these events are not yet clear and appear to be rather complex [107]. In particular, VEGF inhibition causes endothelial cells to reduce NO production. This results in an increased accumulation of platelets, which predisposes to arterial thrombosis [107]. Inhibition of VEGF also promotes proinflammatory gene expression, which contributes to thrombogenesis [107] (Fig. 1).

Vascular endothelial growth factor inhibitors are a family of receptors (VEGFR) containing immunoglobulin-like domains that play a crucial role in endothelial cell migration, survival, and function, and are also critical for the angiogenic response in different types of tumors [108]. TKIs that target these receptors are widely used to treat solid tumors such as NSCLC and metastatic colorectal cancer (mCRC) [109]. In metastatic Renal Cell Carcinoma (mRCC), the survival rate was increased by these inhibitors [109]. Usually, these drugs are not used as a first-line therapy; instead, they are used in later stages and often require long treatment durations [109]. They are therefore associated with an increased risk of vascular toxicity and cardiovascular events such as hypertension, ATE, MI and stroke, which account for 25% to 66% of the fatal events [109]. In particular, inhibitors such as sunitinib and sorafenib may affect coagulation activation, thereby increasing the risk of thromboembolic events and more particular ATE [110]. The incidence of ATE was 1.4%, without being significantly affected by different types of cancer [110]. The mechanism of these events is unknown, but it has been hypothesized that it is related to endothelial dysfunction [74]. At the same time, this effect on endothelial cells increases the risk of hemorrhage [110].

TKIs targeting the BCR-ABL oncogenes are one of the most important agents for the treatment of acute Philadelphia chromosome positive acute lymphoblastic leukemia (Ph+ ALL) and chronic myelogenous leukemia (CML) [74]. We recently demonstrated that molecules analogous to imatinib and nilotinib possess antiplatelet activity [110–112]. Despite their remarkable anti-tumor activity, these inhibitors may induce arterial events, which are likely related to kinase inhibition of endothelial function and activation of the plasma clotting factor [74]. The most frequently observed events are coronary events such as MI and stroke, as well as peripheral vascular events [108]. Imatinib is a first-generation inhibitor and although associated with a low incidence of cardiovascular and arterial thrombotic events, resistance to the therapy is due to ABL-genetic mutations [108]. Therefore, more potent second and third generation inhibitors such as ponatinib, which has the widest spectrum of inhibition of tyrosine kinases, have been developed [74]. Ponatinib is a specific inhibitor that targets the T315I gatekeeper mutation [108]. Although it has shown remarkable anti-cancer activity, it is associated with a very high incidence of acute arterial thrombotic events [108]. Platelet activation appears to be the major ponatinib-associated mechanism of arterial thrombosis [113]. It has been shown that ponatinib causes excessive platelet activation when exposed to a glycoprotein VI agonist [113]. In addition, it increases platelet adhesion by stimulating this mechanism [85]. The use of ibrutinib is strongly associated with an increased risk of arterial fibrillation, with studies showing a 3–4-fold increased risk [114]. (Fig. 1) According to the EPIC study [115], 7.1% of ponatinib-treated patients experienced severe arterial events resulting in treatment discontinuation after just 5.1 months.

In addition, in a phase II study in patients with CML, vascular outcomes were 8.9% at 11 months and 55.9% at 60 months [116]. In the PACE study, the dose-dependent nature of adverse events was highlighted and even when the dose was reduced, the incidence of arterial thrombosis was 20% over five years [116]. Data from the VigiBase pharmacovigilance database also indicates a significantly higher reporting of supraventricular arrhythmias, which in turn translates into an increased risk of stroke [114] (Fig. 2).

#### 4.3.4 Immunotherapy

In recent years, immunotherapy has emerged as a new approach to treat a number of cancers, including non-small cell lung cancer, Hodgkin’s lymphoma, renal cell carcinoma, melanoma, and cervical cancer [117]. By using humanized monoclonal antibodies, this strategy restores the ability of T-cells to recognize and kill tumor cells by blocking important immunosuppressive pathways (e.g., PD-1, PD-L1, and CTLA-4) [117]. In addition, immunotherapy includes other techniques that modify or strengthen the immune system, such as cytokines, radio-labelled antibodies, immunotoxins, and cellular therapies. These treatments are used for various solid and hematological tumors, such as breast, lung, colorectal, prostate, and melanoma [118]. By blocking the typical inhibitory pathways of the immune system, immune checkpoint inhibitors (ICIs) stimulate T-cell activation and restore the anti-cancer response [119]. Through endothelial damage and activation of coagulation factors, this activation may also cause inflammatory responses in healthy tissues such as the vascular endothelium, leading to vasculitis, which is a known risk factor for thrombosis [119]. In addition, T-cell stimulation induces activation of monocytes, increasing the expression of TF, which stimulates the extrinsic thrombogenesis pathway and causes the hypercoagulation observed in some patients with tumors [119].

ICIs can also increase the risk of arterial thrombotic events and promote atherogenesis [120]. When the PD-1 and PD-L1 pathway is blocked, pro-inflammatory cells proliferate in atherosclerotic plaque, promoting inflammation, activation of platelets, and endothelial cells, and ultimately thrombosis [117]. Recent clinical and trial-derived data have provided quantitative estimates of CA-ATE in patients treated with immune checkpoint inhibitors. The reported incidence of arterial thrombotic events ranges from approximately 1%–6%, with cumulative rates of approximately 0.6% at 3 months, 1%–2% at 6 months, and up to 5% at 12 months following ICIs initiation [121]. Although data remain limited, emerging clinical trial–derived and large real-world studies consistently indicate a higher incidence of arterial thrombotic events in ICIs-treated patients compared with non-ICIs-treated cancer populations [122]. Cardiovascular events representing the clinical manifestation of CA-ATE, including MI, ischemic stroke, and coronary revascularization, occurred at rates of 6.55 and 1.37 per 100,000 person-years in ICIs-treated and non-ICIs-treated patients, respectively. 6.55 and 1.37 per 100,000 person-years in the 842 ICPI-treated patients and in the non-ICIs-treated patients, respectively [122]. This suggests a three-fold increased risk of atherosclerotic events [122]. In a large propensity score–adjusted cohort including 2877 ICI-treated patients, exposure to immune checkpoint blockade was associated with a significantly higher risk of arterial thrombotic events compared with non-ICI-treated cancer patients, corresponding to a relative risk of 2.01 (95% CI 1.61–2.51; *p* < 0.001) [120]. Importantly, the excess arterial risk demonstrated a clear time-dependent pattern. No statistically significant increase in ATEs was observed during the first year of therapy when follow-up was restricted to a maximum of nine months (*p* = 0.075) [123]. In contrast, the risk increased by approximately 41% after one year of ICI exposure (*p* = 0.010) and by nearly 97% after four years of follow-up (*p* ≤ 0.001) [123]. In another cohort study of 672 patients with different types of cancer, the cumulative incidence of arterial thrombosis ranged from 1% to 4% to 2% to 5% depending on the type of ICIs and the type of cancer. Patients treated with nivolumab and pembrolizumab had a higher incidence [122]. The cumulative risk of venous thromboembolism of up to 24% in some cases is supported by clinical trial group data, which also suggest a similar risk of arterial and venous thrombosis when ICIs are used [122] (Fig. 2).

We have included a summary table (Table 2) comparing the incidence of arterial thrombotic events across different cancer types and treatment regimens. This table provides a more comprehensive and visually intuitive overview of the available data, enhancing clarity and ease of interpretation for the reader.

**Table 2:** Reported incidence and clinical characteristics of cancer-associated arterial thromboembolic events (ATEs) across tumor types and anticancer therapies.

Cancer Type/Therapy	Reported ATE Incidence/Risk	Clinical Correlation/Notes	References
Lung cancer	8.3% within 6 months	Higher ATE risk; advanced stages increase risk; associated with comorbidities like AF	[21,29,68,121]
Stomach cancer	6.5%	Higher ATE risk; decreased after 1 year	[21]
Pancreatic cancer	5.9%; 4.8-fold increase (Danish study)	Strong predictor of ATE; higher recurrence; mortality risk	[20,68]
Breast cancer	4.7% overall (vs 2.2% controls)	MI more frequent than stroke (3% vs. 2%); advanced stage increases risk; hormonal therapy (TAM, AIs) modifies risk	[20,21,63–73]
Prostate cancer	Not specified	Increased ATE risk with bevacizumab	[20,72,105]
Bladder cancer	Not specified	Higher ATE with VEGF inhibition	[21,22,105]
Non-Hodgkin lymphoma	Not specified	Predominantly venous thrombosis; limited but reported arterial events	[21,22]
AML (Acute Myeloid Leukemia)	2.9% overall within 6 months	Most events in first month; mortality high (~50%)	[122]
Cisplatin-based chemotherapy	Up to 18.1% within 4 weeks	Induces endothelial activation, platelet activation, TF release; arterial events included	[84]
Gemcitabine	2.1–2.2-fold increased risk	Alter platelet function, endothelial damage; may cause thrombotic microangiopathy (0.1–0.3%)	[68–73]
Tamoxifen (TAM)	1.6% increase in premenopausal women; various RORs for arterial events	Platelet activation; reduced antithrombin/C/S; increases FVII, FXI, vWF; risk higher in older and heavier patients	[94]
Aromatase inhibitors (AIs)	Reported incidents range widely (1.1–60.6% overall; ~13% average)	Endothelial dysfunction; increased MI, ischemic stroke, dysrhythmia, valvular dysfunction	[101–104]
Bevacizumab	Not specified	Increased arterial thromboembolic events; dose-dependent; hypertension, ischemic brain injury; tumor type-specific	[72,105]
VEGFR-TKIs (sunitinib, sorafenib)	1.4%	Vascular toxicity; hypertension; MI, stroke; endothelial dysfunction	[106–108]
BCR-ABL TKIs (imatinib, nilotinib, ponatinib)	Low (imatinib); very high (ponatinib)	Arterial thrombosis (MI, stroke, peripheral); platelet activation major mechanism; dose-dependent	[72,106,111–113,123]
ICPIs (nivolumab, pembrolizumab, others)	1–5% cumulative incidence; 3-fold increased risk vs. non-ICPI	Induce endothelial damage, coagulation activation, atherogenesis	[115–117,119,123]

Note: Abb.: Myocardial Infraction (MI), Tissue factor (TF), ROR (Reporting Odds Ratio), BCR-ABL (Breakpoint cluster region-Abelson).

## 5 Conclusions

Cancer-associated arterial thromboembolism (CA-ATE) represents an increasing clinical challenge and a leading cause of cancer-related morbidity and mortality. Although VTE has been extensively studied, there remain significant gaps in the understanding of ATE, despite its often abrupt and severe onset. The pathogenesis of arterial thrombosis can be conceptualized through Virchow’s triad, with mechanisms such as platelet hyperactivity, TF overexpression, elevated vWF, and NETs overproduction contributing to a prothrombotic environment.

Risk factors involve patient characteristics, cancer type and stage, as well as anti-cancer therapies. Age, comorbidities such as hypertension and diabetes, and lifestyle factors (e.g., smoking, obesity) increased risk. Certain malignancies, including pancreatic, lung, and hematological cancers, are particularly associated with higher ATE incidence. Cancer treatments—including cisplatin-based chemotherapy, hormone therapy with tamoxifen, VEGF inhibitors, and various tyrosine kinase inhibitors (TKIs)—further amplify thrombotic risk.

Future perspectives include the need for dedicated studies on the mechanisms and predictors of CA-ATE to improve early identification and prevention strategies. Research should also focus on optimizing pharmacological prophylaxis, potentially incorporating antiplatelet agents or novel anticoagulants, while balancing the risk of bleeding. Evaluating the thrombotic profiles of newer cancer therapies and personalizing prevention based on patient and tumor characteristics represent key avenues for future clinical management.

In routine cancer care, early identification of patients at increased risk of thrombosis associated with cancer may enable early initiation of preventive measures. By identifying specific risk factors, clinicians may be able personalized thromboprophylaxis to a patient with cancer. Adverse events can be reduced by specific anticoagulant treatment to the individual risk profile of each patient.

## Data Availability

Since this study did not generate or analyze any datasets, data sharing is not applicable to this article.
